# Predictors of premarital cohabitation timing among young women in Ethiopia: insights from the 2016 demographic and health survey using a shared frailty model

**DOI:** 10.3389/fgwh.2024.1327219

**Published:** 2024-10-03

**Authors:** Teshome Demis Nimani, Eyob Eshete Tadese, Fikadu Wake Butta, Zinabu Bekele Tadese

**Affiliations:** ^1^ Department of Epidemiology and Biostatistics, School of Public Health, College of Medicine and Health Science, Haramaya University, Harar, Ethiopia; ^2^ Department of Nursing, College of Health Science, Mettu University, Mettu, Ethiopia; ^3^ Department of Health Informatics, School of Public Health, College of Medicine and Health Science, Mettu University, Mettu, Ethiopia; ^4^ Department of Health Informatics, School of Public Health, College of Medicine and Health Science, Samara University, Samara, Ethiopia

**Keywords:** premarital cohabitation, youth women, shared frailty, Ethiopia, time

## Abstract

**Introduction:**

Most new HIV infections occur in sub-Saharan Africa due to premarital, likely experiences of abuse, exploitation, intimate partner violence, murder, and suicide. Transient nature of the relationship, cohabiting young females are frequently at risk for poor mental health following disagreements. This study's aim was to predictors of premarital cohabitation timing among young women in Ethiopia.

**Method:**

Secondary data from the 2016 Ethiopian Health and Demographic Survey was analyzed. The study comprised a weighted sample of 6,142 young women. A weighted descriptive analysis of graphs, frequency tables, medians, and percentiles was performed to describe the study participants. The Akaike information criteria were used to choose the best-shared frailty model for the data. Final measures of effect size included the adjusted hazard ratio, both of which had a *p*-value of less than 0.05.

**Result:**

Premarital cohabitation was reported to have a median age of 16 years (IQR, 15–18 years). Woman's age (AHR = 0.795; 95% CI: 0.761–0.868) was one of the independent predictors of time to premarital cohabitation. For primary, secondary, and higher education, respectively, 0.733 (95% CI: 0.607, 0.959) and 0.610 (95% CI: 0.589, 0.632) were seen among women who can read and write (AHR = 0.896; 95% CI: 0.872, 0.920). Women with access to the media (AHR = 0.722, 95% CI: 0.510, 0.963).

**Conclusion:**

The most important idea is that educational level, access to media, age, and literacy are the most significant factors for the time-to-premarital cohabitation rate.

## Introduction

Cohabitation is used to describe unmarried couples who are in a relationship and live together. It applies to opposite-sex individuals (1). Two people residing together as though they were a married couple is known as cohabitation.

Around 1.5 million couples cohabited globally in 1996; 3.6 million couples cohabited globally in 2021 (2). In the second half of the 20th century, cohabitation became highly prevalent, especially among young women between the ages of 15 and 24, notably in nations like France, Sweden, Denmark, and the United Kingdom. with high levels before 1960: El Salvador (34.2%) and Guatemala (29.7%) (3). Cohabitation increased starting in 1960, with countries such as Colombia (13.5%), Peru (20.9%), Guatemala (37.2%), and Venezuela (44.4%) (4). Results employing microdata samples Integrated Public Use Microdata Series (IPUMS) International data from the Latin American census show rising trends in cohabitation among women in Latin American nations who are 25 years of age and older. In Venezuela, for example, the proportion of couples increased by 15% in 10 years, from 37% in 1990 to 52% in 200 (4). The prevalence of cohabitation was the greatest in Central Africa (21.7%) and the lowest in West Africa (6.2%). Whereas cohabitation rates in East and Southern Africa were 11.7% and 10.4%, respectively (5).

Nowadays, cohabiting couples primarily in sub-Saharan Africa are responsible for new HIV infections (6). Cohabiting college students frequently have unprotected sex, which increases the risk of STDs and HIV/AIDS diseases. Develop unwanted pregnancies and frequently abort their children, resulting in uterine damage and death, cohabiting couples accounted for the majority of non-marital births during the 1990s (7, 8). Women who cohabitate early in their lives are more likely to develop breast cancer (9). Cohabiting women died at a higher rate than married women (10). Moral and theological degradation, while the effects of cohabitation included mortality, dropout from school, low academic performance, and health or social difficulties (11).

According to a study conducted in 19 African countries, 18% of cohabiting respondents were female, and 54% of them were addicted to alcohol (12–15). A comparative analysis reveals that cohabitated women die more frequently from cardiovascular, respiratory, gastrointestinal, alcoholic, and accident-related causes of mortality compared to married individuals (16). Cohabitation resulted in very likely experiences of abuse, exploitation, intimate partner violence, murder, and suicide (17–19). Due to the frequent lack of commitment and transient nature of the relationship, cohabiting female youths are frequently at risk for poor mental health following disagreements (20, 21).

Premarital cohabitation results in divorce around the world. There is disagreement among scholars as to why premarital cohabitation has been associated with increased divorce rates. Researchers disagree on whether there is a longer-term relationship between premarital cohabitation and divorce. In the past, premarital cohabitation has been linked to increased divorce rates (22–24). In premarital cohabitation, according to the findings of the systematic review, these life events may cause a shift in priorities, which may impact lifestyle choices and present opportunities for improving health choices such as food and physical activity (25). There was evidence that cohabiting Marijuana usage among women was more prevalent than other drugs regularly (26). Early marriage and adolescent cohabitation are both associated with an increased likelihood of having a child (27, 28). African traditional practices are challenged by cohabitation, which also erodes the fundamental ideals and standards of marriage on that continent (29).

The median age of premarital cohabitation in South Africa is 15–24 years old (30), in Bangladesh, between the ages of 15 and 19 (31), surveys on health and demographics conducted in Sub-Saharan Africa (DHS), 20–24 years (5).

There are numerous factors contributing to the high prevalence of premarital cohabitation in the world. This includes living in a rural area, which increases (5, 32), Religion being Catholic increases the likelihood of cohabitation (5, 32). Having wealth is negatively associated with cohabitation (5, 30, 32). Having media exposure also reduces premarital cohabitation (30), As women's educational attainment level increases, the likelihood of premarital cohabitation decreases (5, 30, 32), and having occupation increases the likelihood of premarital cohabitation (5, 32). Behavioral factors Smoking cigarettes and alcohol consumption also affect cohabitation; a study conducted in Thailand found that smokers and current regular drinkers were all more likely to cohabit (6).

However, previous studies missed major factors like literacy, age, tobacco use, smoking others, hearing about HIV/AIDS, ever hearing about STDs and region, and there is no nationwide evidence about the timing of premarital cohabitation. Even though its evolution still needs to be studied, the current study aimed to assess predictors of premarital cohabitation timing among young women in Ethiopia: Insights from the 2016 Demographic and Health Survey using a shared frailty model.

## Method

### Study design and period

A community-based cross-sectional survey was conducted from January 18, 2016, to June 27, 2016, in Ethiopia.

### Study area

Ethiopia was the study's location. A sub-Saharan African nation with the second-largest population in Africa in 2016 at 102 million people. The bulk of women—78%—were rural residents (37).

### Study participants

The study included all young women (15–24 years old) found in the selected clusters at least one night before the data collection period of January 18, 2016 to June 27, 2016. Taking youth age-women (15–24 years) of Ethiopia in place of the source population, youth age women living in selected clusters as the study population, as well as the young women (15–24 years old) discovered in the 2016 Ethiopian Demographic Health Study (EDHS) enumeration areas at least one night before data collection as per the sample population (33).

### Sampling technique and Sample size determination

**Figure 1:**
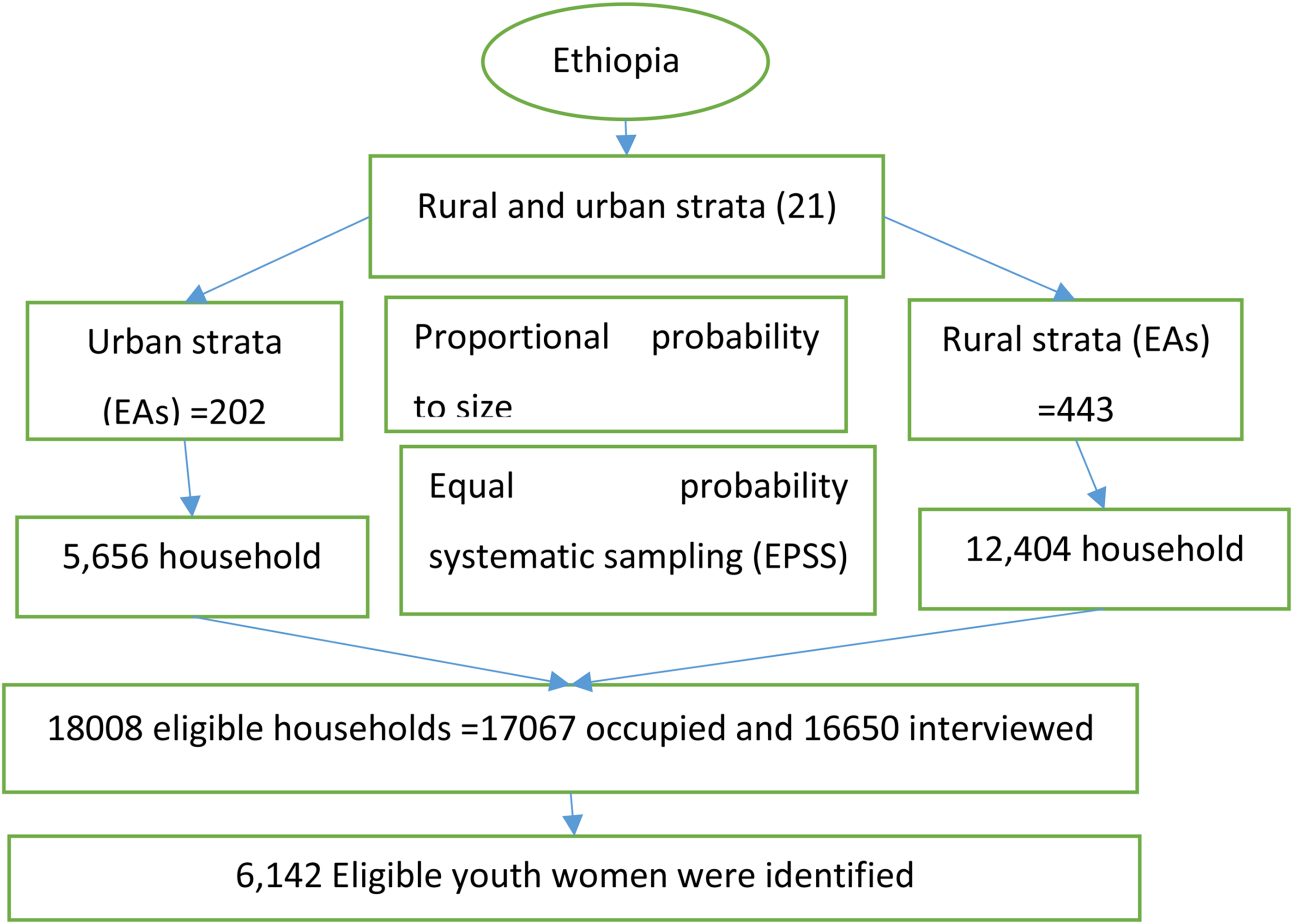
Sampling procedure of time to premarital cohabitation among Ethiopian youth women in DHS 2016.

Census enumeration areas (EAs) served as the sampling units for the first stage of the stratified two-stage cluster sampling design that was used to select the 2016 DHS sample. An updated list of all the households in each EA was used to select a sample of homes for the second stage. For the sample, 18,008 households in total were chosen, of which 17,067 were inhabited. 16,650 of the inhabited homes were successfully contacted for interviews, resulting in a 98% response rate. 16,583 eligible women from the households surveyed were selected for one-on-one interviews. 15,683 women participated in interviews, resulting in a 95% response rate (33). After their exclusion from the data, the effective women out of the youth (15–24) sample size became 6,142. The schematic representation is shown in Figure 1 below (34, 35).

### Data quality control

After all, after being finalized in English, the surveys were translated into regional tongues (Amarigna, Tigrigna, and Oromifa) and pretested at Bisheftu. Computer-assisted personal interview data collection system was carried out to collect data by trained EDHS data collectors and mobile version CSPro software was used for entering and capturing the data (33).

The data collectors and study participants were blind to the study hypothesis since the analysis was considered later. A data extraction checklist was prepared and data was extracted using Stata version 17.

### Data source

Secondary data from the 2016 EDHS were used in this investigation. After obtaining a letter from the DHS approving its usage, the data set was retrieved from the website https://dhsprogram.com. Using a data extraction tool, variables were taken out of the EDHS 2016 individual women's data collection.

### Outcome and independent variable

The study's outcome variable was the length of time (measured in years) that elapses between a woman's commencement of cohabitation and the conclusion of the data collection period. Factors related to age, region, religion, and place of residence are all considered sociodemographic. Socioeconomic variables encompass the wealth index, media exposure, women's educational attainment, literacy, and work position. Alcohol abuse, tobacco usage, other smoking, and cigarette smoking are examples of behavioral factors. Factor connected to knowledge: You have probably heard about HIV/AIDS and STDs.

### Measurement of variables

The dependent variable, time to premarital cohabitation measured in years, was taken from age at first premarital cohabitation as an otherwise censored event. For analysis, those women who cohabited had an event code of 1 (success), and those who did not cohabit had a code of 0 (censored). Independent variables. The respondent's education was categorized into no education, primary, secondary, and higher education, and no education was taken as a reference. The respondent's occupation is coded as “not working” and has a working reference number of “working.” The index was classified as (poor, middle, and rich) by taking the poor as the comparison group. Mass media exposure (yes/no), and Ever heard about HIV/AIDS and STDs (yes/no), literacy coded as “can read and write but cannot read and write tobacco use, smoking cigarettes, alcohol consumption, and smoking other” (yes/no)?

### Operational and concept definition

**Media exposure;—**The frequency with which respondents watched television, listened to the radio, or read a newspaper was inquired about. Those who had exposure to one of them at least once a week are considered to be regularly exposed to media (35).

**Wealth index:—**An amalgamated indicator of a household's overall standard of life is the wealth index. Easy-to-collect information on a household's ownership of specific items, such as TVs and bicycles; building materials; and kinds of water access and sanitation facilities, produced using a statistical process known as principal components, is used to calculate the wealth index. Through analysis, the wealth index plots each household's relative wealth on a continuous scale. DHS divides all families surveyed into five wealth quintiles to examine the impact of wealth on a range of demographic, health, and nutrition metrics (36).

**Event:—**premarital cohabitation (living together as a couple before marriage) coded as event =1.

**Censored:—**there is no status of premarital cohabitation (not living together as a couple before marriage) coded as censored =0.

### Data analysis and management procedure

The necessary data from individual records of women from the 2016 DHS was kept, cleaned, coded, merged, and appended using STATA version 17, and a model was run using R-software version 4.1.3. According to the DHS guidelines, the missing value imputed for categorical variable mode and for quantitative variable normally distributed used mean unless median were imputed and above 15% missed was left out the variable from analysis. Descriptive metrics like medians, percentiles, graphs, and frequency tables are used to characterize the study participants once the data has been extracted, cleaned, and weighted. The Kaplan–Meier (K–M) technique was used to determine the median time to premarital cohabitation, and the log-rank test was used to evaluate categorical predictor variables between groups. The time to premarital cohabitation among young women in Ethiopia was assumed to be constant in the same clusters, and enumeration areas or clusters were used as a random effect for predictors of the time to premarital cohabitation, resulting in a parametric shared frailty model since the data were correlated at the cluster level. The model with the lowest AIC and BIC values was chosen as the most efficient one. Cox-Snell residuals were used to assess the appropriateness of the model.

### Gamma frailty distribution

The gamma distribution better model than others in the case of large data due to this reason we discuss this model, The gamma distribution is frequently used, for instance, as a mixture distribution (37, 38). It fits into survival models quite well computationally, as it is simple to generate the formulas for any number of occurrences. This is because the Laplace transform's derivatives are straightforward. The gamma frailty distribution's straightforward interpretation, adaptability, and mathematical tractability have made it a popular choice for parametric intra-cluster dependence modeling (39, 40). To identify the model, we limit the variance to be finite and the expectation of the frailty to equal one, requiring the estimation of just one parameter. As a result, the one parameter gamma distribution is the frailty *Z* distribution. The relevant density function and Laplace transformation of the gamma distribution, subject to the limitation, are provided by (41):

$$f_{z}(Z)=\frac{Z_{i}^{-1+\left(\frac{1}{\theta }\right)}}{\theta^{\frac{1}{\theta }}\Gamma \left(\frac{1}{\theta }\right)}\exp\;\left(\frac{-Zi}{\theta }\right),\;\theta >0$$

Where Г (.) is the gamma function, it corresponds to a Gamma Distribution Gam (µ, *θ*) with µ fixed to 1 for identifiability, and its variance is *θ*. The associated Laplace transform is: -

$$L(u)={\left(1+\frac{u}{\theta }\right)}^{-\theta },\;\theta >0$$

Keep in mind that there is heterogeneity if *θ* > 0. Therefore, large values of *θ* indicate stronger associations within groups as well as a greater degree of variety among them. The gamma frailty distribution's conditional survival and hazard function is provided by (41):

$$S_{\theta }(t)=[1-\theta \ln\;(S(t)){]}^{\frac{-1}{\theta }}$$

$$h_{\theta }(t)=h(t)[1-\theta \ln\;(S(t)){]}^{-1}$$

Where *S* (*t*) and h (*t*) are the baseline distributions’ hazard and survival functions, respectively. Kendall's Tau for the Gamma distribution (42), which, in the multivariate instance, quantifies the correlation between any two event times from the same cluster. It is a general indicator of reliance that is unaffected by changes in the time scale or the frailty model being applied. Kendall's is used to measure the associations among group members and can be obtained as follows:

$$\tau =\frac{\theta }{\theta +2}\;\epsilon (0,1)$$

### Ethical consideration

Ethical clearance was accessed for the DHS dataset by using the DHS website (http://www.dhsprogram.com) after submitting the proposal title, justification, and objective. The data was handled properly and kept confidential only by giving it to those who are mentioned in the DHS application letter as co-authors.

## Result

### Participant characteristics

This study included a weighted total of 6,142 young women. The time to premarital cohabitation was an interest of this research paper on total young women. 3,500 (57%) of them cohabitated; 2,643 (43%) of them did not cohabitate until the end of the data collection. Different covariate characteristics are displayed in Table 1. 4,675 (76%) of the 6,142 female youths lived in rural areas, while 1,467 (24%) lived in urban areas.

**Table 1:** Respondents’ characteristics with their status for predictors of premarital cohabitation timing among young women in Ethiopia: insights from the 2016 demographic and health survey using a shared frailty model.

Variables	Categories	Frequency	Status	
			Censored	Event
Place of residence	Urban rural	1,467 (24%)	1,087 (74%)	381 (26%)
		4,675 (76%)	2,413 (52%)	2,262 (48%)
Religion	Orthodox	2,640 (43%)	1,543 (58%)	1,097 (42%)
	Catholic	53 (1%)	33 (62%)	20 (38%)
	Protestant	1,487 (24%)	998 (67%)	490 (33%)
	Islamic	1,883 (31%)	904 (48%)	979 (52%)
	Other	79 (1%)	22 (28%)	58 (72%)
Educational status	No education	1,230 (20%)	341 (28%)	889 (72%)
	Primary education	3,333 (54%)	1,990 (60%)	1,342 (40%)
	Secondary & above	1,580 (26%)	1,168 (74%)	412 (26%)
Literacy	Cannot read & write	2,224 (36%)	823 (37%)	1,401 (63%)
	Can read & write	3,919 (64%)	2,677 (68%)	1,242 (32%)
Wealth index	Poor	2,026 (33%)	856 (42%)	1,170 (58%)
	Middle	1,114 (18%)	588 (53%)	525 (47%)
	Rich	3,003 (49%)	2,055 (68%)	948 (32%)
Smoking cigarettes	No	6,116 (99%)	3,495 (57%)	2,621 (43%)
	Yes	26 (1%)	4 (15%)	22 (85%)
Tobacco use	No	6,140 (99%)	3,499 (57%)	2,641 (43%)
	Yes	1 (1%)	0 (0%)	1 (100%)
Smoking other	No	6,141 (99%)	3,400 (55%)	2,641 (45%)
	Yes	1 (1%)	0 (0%)	1 (100%)
Alcohol consumption	No	4,171 (68%)	2,404 (58%)	1,767 (42%)
	Yes	1,971 (32%)	1,096 (56%)	875 (44%)
Occupation status	Has no occupation	3,451 (56%)	1,962 (57%)	1,490 (43%)
	Has occupation	2,691 (44%)	1,538 (57%)	1,153 (43%)
Ever heard about STDs	No	377 (6%)	188 (50%)	189 (50%)
	Yes	5,766 (94%)	3,312 (57%)	2,454 (43%)
Ever heard about HIV/AIDS	No	393 (6%)	191 (49%)	202 (51%)
	Yes	5,750 (94%)	3,309 (58%)	2,441 (42%)
Media exposure	Has no media	5,989 (98%)	3,383 (56%)	2,606 (44%)
	Has media	154 (2%)	117 (76%)	3 (24%)

The wealth index of a family was categorized as having a low, middle, or high income. It is reported that 2,026 (33%), 1,114 (18%), and 3,003 (49%), respectively, women lived in poor, middle, and rich households. More than half of the women, 4,171 (68%), have no jobs. 1,580 (26%) of the total women attained secondary and above education, 3,333 (54%) of the youth's women attained primary education, and 1,230 (20%) of them were uneducated. Of the total number of young women, 2,640 (43%) were Orthodox, and 53 (1%) were Catholic. Muslim, 1,883 (31%) were Protestant, 1,487 (24%) were Catholic, and 79 (1%) of them were from other religions. Furthermore, 1,971 (32%) of the young women had experienced drinking alcohol. Concerning exposure to mass media, 5,989 (98%) of the women had no access, and 154 (2%) of them did.

### Median survival time of premarital cohabitations

Non-parametric survival analysis is very important to visualize the survival of time-to-premarital cohabitation of young women in Ethiopia was 16 with a median interquartile range (15, 18) for each categorical variable with respective categories. Regarding residence area and religion categories, their median survival time was 17. Similarly, the median survival time of those wealth index poor and middle 16 and their counterparts 17 (IQR: 15, 19) summarized in Table 2.

**Table 2:** Median survival time for time to premarital cohabitation among youth women in Ethiopia 2016 demographic and health survey.

Variable	Categories	Median time Median IQR	Chi-square value	Log-rank test
Overall		16 [15, 18]		
Residence area	Urban	17 [15, 18]	142.27	<0.001
	Rural	16 [15, 18]		
Religion	Orthodox	17 [15, 19]	46.38	<0.001
	Catholic	16 [15, 17]		
	Protestant	17 [15, 18]		
	Islam	16 [15, 18]		
	Other	16 [14, 18]		
Educational status	No education	16 [14, 17]	283.43	<0.001
	Primary education	18 [15, 18]		
	Secondary & above	18 [16, 20]		
Literacy	Cannot read & write	16 [14, 17]	205.10	<0.001
	Can read &write	17 [15, 19]		
Wealth index	Poor	16 [15, 17]	160.78	<0.001
	Middle	16 [15, 18]		
	Rich	17 [15, 19]		
Smoking cigarettes	No	16 [15, 18]	0.80	0.3712
	Yes	17 [16, 19]		
Tobacco use	No	16 [15, 18]	2.21	0.1369
	Yes	15 [14, 16]		
Occupation status	Has no occupation	16 [15, 18]	22.78	<0.001
	Has occupation	17 [15, 18]		
Ever heard about STDs	No	16 [15, 18]	0.84	0.3600
	Yes	16 [15, 18]		
Ever heard about HIV/AIDS	No	16 [15, 18]	1.56	0.2116
	Yes	16 [15, 18]		
Smoking others	No	16 [15, 18]	0.9	0.3422
	Yes	16 [15, 17]		
Alcohol consumption	No	16 [15, 18]	7.13	0.0076
	Yes	17 [15, 18]		
Media exposure	Has no media	16 [15, 18]	25.31	<0.001
	Has media	19 [17, 21]		

### Parsimonious model selection

#### Cox proportional hazard model

The multivariable Cox model contained all twenty-two predictor variables that were significant at 0.2 *p*-values in the bivariable analysis. Ten, the proportional hazard assumption of the time to first birth data was assessed using the Schoenfeld test. The strong association between time and premarital cohabitation (Table 3) violated the proportional hazard assumption in both the global and rank tests; hence, the Cox model was ruled out for this set of data. Because there isn't a predictor variable in the stratified Cox model that satisfies the proportional hazard assumption, the model is likewise incorrect for these data. The problem of deciding which function of survival time to include in the model also challenges another alternative time-varying Cox model. But about our data.

**Table 3:** Proportional hazard assumption predictors of premarital cohabitation timing among young women in Ethiopia: insights from the 2016 demographic and health survey using a shared frailty model.

	chi2	df	Prob > chi2
Global test	89.65	18	<0.001

### Multivariable analysis

All the factors that are significant in the univariate analysis at a 25% level of significance were included in the multivariable PH and AFT models of the exponential, Weibull, log-logistic, exponential, Gompertz, and log-normal distributions for the time-to-premarital cohabitation data. The generalized gamma and exponential distributions were eliminated from the model. As the word “frailty” has no real significance. The AIC was employed to compare the effectiveness of various models. This is the model selection criterion that is most frequently used. An AIC-based model with the lowest value was favored. So, the Weibull gamma frailty model (AIC = −3,171.108) is determined to be the best of the options provided for the time-to-premarital period when all factors that are significant in the univariate analysis are included. As a result, every information that has ever been heard regarding tobacco usage, HIV/AIDS, cigarette smoking, and other types of smoking was left out. The covariates of women's age, place of residence, religion, level of education attained by women, region, media exposure, and employment status were retained in the final model. Four parametric models with gamma and inverse Gaussian frailty and the corresponding AIC values are displayed in Table 4.

**Table 4:** Model comparison for predictors of premarital cohabitation timing among young women in Ethiopia: insights from the 2016 demographic and health survey using a shared frailty model.

Model	Frailty	Likelihood ratio	df	AIC	BIC
Gompertz	Gamma	1,484.486	22	−2,924.971	−2,794.514
Log-logistic	Gamma	1,568.935	22	−3,093.869	−2,963.413
Weibull with HR	Gamma	1,607.054	22	−3,171.108	−3,039.651
Weibull with AFT	Gamma	1,607.054	22	−3,170.108	−3,039.651
Log-normal with AFT	Gamma	1,543.932	22	−3,043.863	−2,913.406

Table 4 Model comparison for predictors of premarital cohabitation timing among young women in Ethiopia: insights from the 2016 demographic and health survey using a shared frailty model.

### Weibull gamma frailty model result

It is assumed that the frailty in this model has a variance equal to theta (*θ*) and a mean of 1. Theta's (*θ*) calculated value is 0.019. If there is no variance (*θ* = 0), it means that the frailty component doesn't add anything to the model. Table 5 below displays the results of a likelihood ratio test for the hypothesis *θ* = 0, which shows a significant *P*-value of 0.000 and a chi-square value of 19.56 with one degree of freedom. This suggested that the frailty element significantly influenced the model. Additionally, the predicted value of the corresponding Kendall's tau (*τ*), which gauges dependence within clusters (countries), is 0.009. In the Weibull gamma frailty model, the form parameter has an estimated value of 8.59 (*P*). Because the value is greater than unity, it indicates that the hazard function grows to a maximum point before decreasing, demonstrating the unimodal structure of the function. The confidence intervals for all relevant factors (youth age, educational attainment, literacy, and media exposure) in Table 5 do not include one at the 5% significance level. This demonstrated that they play a major role in predicting how long Ethiopian young women will live together before getting married. Consequently, the hazard probability of premarital cohabitation drops by 20% with increasing female age (HR = 0.795; 95% CI: 0.761, 0.868). The survival time of premarital cohabitation of women is lower by rates of 0.733 (95% CI: 0.607, 0.959) and 0.610 (95% CI: 0.589, 0.632) for primary, secondary, and above education, respectively; in other words, women who have attended at least primary school have decreased the rate of premarital cohabitation more than those who have no education. For women who can read and write, the rate of premarital cohabitation was decreased by 10% compared with women who cannot read and write (HR = 0.896; 95% CI: 0.872, 0.920). For a woman who has media access, the rate of premarital cohabitation decreased by 22% compared with a woman who has no media access (HR = 0.722, 95% CI: 0.510, 0.963).

**Table 5:** Gompertz gamma frailty model result for predictors of premarital cohabitation timing among young women in Ethiopia: insights from the 2016 demographic and health survey using a shared frailty model.

Factors	Hazard Ratio	Standard error	Z-cal	*p*-value	[95% CI]
Age	0.795	.0016394	−125.21	<0.001	[0.761, 0.868]
Religion ref (orthodox)					
Catholic	0.978	.0210964	−1.01	0.313	[0.938, 1.021]
Protestant	1.058	.0217114	2.75	0.371	[.916, 1.101]
Islamic	0.878	.0530553	−2.15	0.132	[0.780, 1.988]
Other	0.953	.085053	−0.53	0.593	[0.800, 1.136]
Residence area ref (urban)					
Rural	1.036	.0133292	2.76	0.060	[.910, 1.063]
Educational status ref (no education)					
Primary education	0.733	.0134095	−4.85	<0.001	[0.607, 0.959]
Secondary & above	0.610	.0109787	−27.46	<0.0010.000***	[0.589, 0.632]
Literacy ref (cannot read & write)					
Can read and write	0.896	.0122736	−8.01	<0.001	[0.872, 0.920]
Wealth index ref (poor)					
Middle	0.951	.0119927	−3.94	0.113	[0.928, 1.075]
Rich	0.828	.0114672	−13.60	0.102	[0.806, 1.051]
Occupation status ref (has no occupation)					
Has occupation	0.074	.0120093	6.38	0.091	[0.051, 1.098]
Media exposure ref (has no media access)					
Has media access	0.722	.0135179	−4.56	<0.001	[0.510, 0.963]

θ=0.019p=8.59τ=0.009LR test of theta = 0: chibar2 (01) = 19.54 Prob ≧chibar2 = 0.000 Log likelihood = 1,568.93 Ref = reference group ***significant at 95% level of confidence CI, confidence interval.

### Cox- snell residuals plots

**Figure 2:**
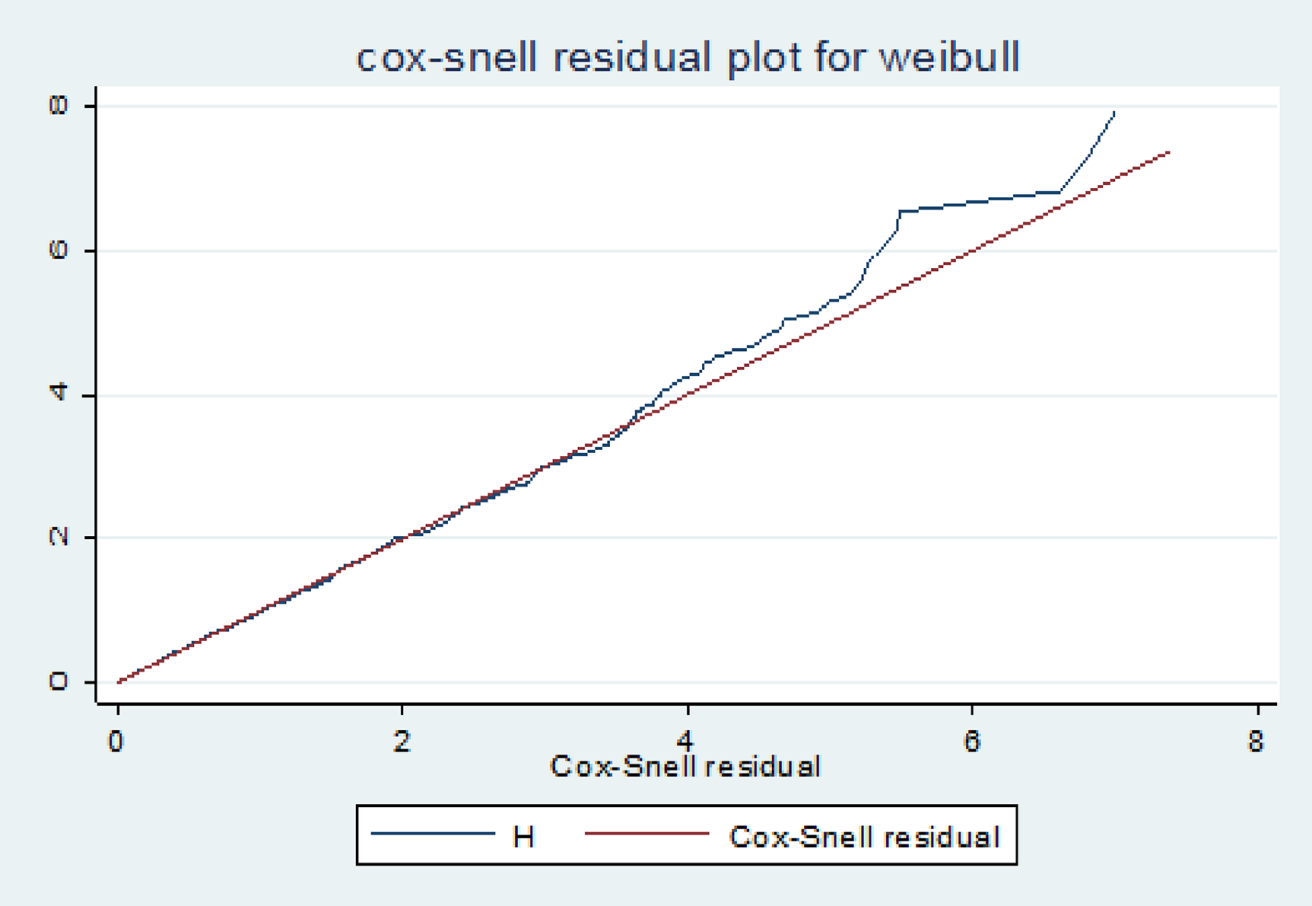
Cox-Snell residual plot for predictors of premarital cohabitation timing among young women in Ethiopia: insights from the 2016 demographic and health survey using a shared frailty model.

One method to look into how well the model fits the data is to use the Cox-Snell residuals. Figure 2 below shows the plot for the fitted model of residuals for Weibull to our data using cumulative hazard functions and maximum likelihood estimation. Plotting the cumulative hazard function of residuals against Cox-Snell residuals should resemble a roughly straight line with a slope of 1 if the model fits the data. The graphic indicates that the Weibull baseline distribution is acceptable for the time-to-premarital cohabitation data set because it cuts through its origin in a straight line.

## Discussion

This study examined the timing of premarital cohabitation among young women aged 15–24 in Ethiopia and modelled factors affecting it using a parametric shared frailty analysis method. The study revealed that the age of youths, educational status, literacy, and media exposure were the most significant factors.

According to the current study, premarital cohabitation occurred at a median age of 16, with an IQR of 15–19 years. This result is consistent with the results from Zimbabwe, Bangladesh, and Ethiopia, where the median age at premarital cohabitation was 15–24 years in Zimbabwe, 15–19 years in Nigeria, and 18 years in Ethiopia (30–32). This may be a result of the high frequency of sexual activity in these nations (43). Another explanation for this resemblance could be that most sub-Saharan countries have fewer educational opportunities for girls because most people live in rural areas (44), It compels them to seek out financial and social assistance (45).

Women's education and premarital cohabitation were inversely associated in this study. This result was consistent with research conducted in South Africa (5), Ethiopia (32), and Bangladesh (30). One explanation for the inverse relationship between educational achievement and premarital cohabitation may be that early marriage and sexual experience are less common among females enrolled in and retained in secondary education, and that girls’ understanding of reproductive health issues is raised (46).

The timing of premarital cohabitation was found to be significantly influenced by one's access to mass media. This could be because if women are aware of the consequences of cohabitation, they are less likely to engage in it (47).

The finding shows that the age of the youth is another important covariate for time-to-premarital cohabitation. This might be because age increases gradually with marriage (48).

Literacy was found to have a significant effect on the timing of premarital cohabitation. The possible justification for this result was that women who can read and write are more aware of the consequences of cohabitation through different media and are less likely to engage in it compared with those who cannot read and write (31).

It is important to consider the various limitations when interpreting the study's conclusions. To start with, self-report bias may be present in the study because it is based on self-reported data (recall and social desirability bias). One possibility is that the age at which people cohabitated for the first time was not fully reported. Additionally restricted to background characteristics were the predictors used in the analysis. Time-to-premarital cohabitation may be greatly impacted by additional factors, such as parental education, that were not examined in the investigation. Since the only factor taken into account in the study was present religion, some factors, like religion, are time-varying to predict the outcome.

Even with these drawbacks, the results point to a few critical elements that are probably important motivators and at a much-advanced level for young women's time to premarital cohabitation. This study's ability to estimate the timing of premarital cohabitation among young Ethiopian women aged 15–24 through the use of population-based and nationally representative data makes the findings broadly applicable to young women in sub-Saharan Africa and other developing nations. The finding's use of statistical analysis to identify the best model for the data under consideration is another crucial strength.

## Conclusion

The median age of premarital cohabitation for Ethiopian women was 16 years old, according to this study.The study result showed that the educational level of women, access to media, age, and literacy were the most significant factors for time-to-premarital cohabitation. Those who have attended at least primary school have decreased the rate of premarital cohabitation more than those who have no education. For women who can read and write, the rate of premarital cohabitation was lower compared with women who cannot read and write. As the age of women increases, the hazard rate of premarital cohabitation decreases. For a woman who has media access, the rate of premarital cohabitation decreased compared with a woman who has no media access.

The Ministry of Women and Children's Affairs will launch initiatives to educate the public and uphold the legality of marriage to decrease early premarital cohabitation. By expanding access to rural, predominant areas, the Ministry of Education suggested keeping women in education until at least secondary school and beyond. To make the most of the media, the Ministry of Health should expand its reach and highlight the negative effects of cohabitation before marriage. Scholars ought to carry out investigations that encompass familial elements and explore the variables that could impact the onset of cohabitation before marriage, there was heterogeneity in time for cohabitation before marriage among young at the Ethiopian level. To identify the hotspot area, researchers need to study the spatial distribution of cohabitation before marriage.

## Data Availability

Publicly available datasets were analyzed in this study. This data can be found here: website https://dhsprogram.com. Further inquiries can be directed to the corresponding author.
